# The Deep-Sea Polyextremophile *Halobacteroides* *lacunaris* TB21 Rough-Type LPS: Structure and Inhibitory Activity towards Toxic LPS

**DOI:** 10.3390/md15070201

**Published:** 2017-06-27

**Authors:** Flaviana Di Lorenzo, Angelo Palmigiano, Ida Paciello, Mateusz Pallach, Domenico Garozzo, Maria-Lina Bernardini, Violetta La Cono, Michail M. Yakimov, Antonio Molinaro, Alba Silipo

**Affiliations:** 1 Department of Chemical Sciences, University of Naples Federico II, 80126 Naples, Italy; flaviana.dilorenzo@unina.it (F.D.L.); mateusz.pallach@gmail.com (M.P.); molinaro@unina.it (A.M.); 2 CNR-Istituto per i Polimeri, Compositi e Biomateriali IPCB–Unità di Catania, 95126 Catania, Italy; angelo.palmigiano@tiscali.it (A.P.); dgarozzo@unict.it (D.G.); 3 Department of Biology and Biotechnology "Charles Darwin", Sapienza-University of Rome, 00185 Rome, Italy; ida_paciello@libero.it (I.P.); maria.bernardini@uniroma1.it (M.-L.B.); 4 Marine Molecular Microbiology & Biotechnology, CNR-Institute for Coastal Marine Environment, 98122 Messina, Italy; violetta.lacono@iamc.cnr.it (V.L.C.); michail.yakimov@iamc.cnr.it (M.M.Y.); 5 Immanuel Kant Baltic Federal University, 236040 Kaliningrad, Russia

**Keywords:** extremophiles, halophiles, *Halobacteroides*, deep-sea hypersaline anoxic basins (DHABs), lipopolysaccharide (LPS), innate immunity, lipid A, core oligosaccharide

## Abstract

The structural characterization of the lipopolysaccharide (LPS) from extremophiles has important implications in several biomedical and therapeutic applications. The polyextremophile Gram-negative bacterium *Halobacteroides* *lacunaris* TB21, isolated from one of the most extreme habitats on our planet, the deep-sea hypersaline anoxic basin *Thetis*, represents a fascinating microorganism to investigate in terms of its LPS component. Here we report the elucidation of the full structure of the R-type LPS isolated from *H. lacunaris* TB21 that was attained through a multi-technique approach comprising chemical analyses, NMR spectroscopy, and Matrix-Assisted Laser Desorption Ionization (MALDI) mass spectrometry. Furthermore, cellular immunology studies were executed on the pure R-LPS revealing a very interesting effect on human innate immunity as an inhibitor of the toxic *Escherichia coli* LPS.

## 1. Introduction

In the last 30 years, the fascinating world of microorganisms able not only to survive but also to proliferate at extreme conditions has attracted researchers in several multidisciplinary scientific fields. Spanning through the three domains of life, such microorganisms, known as extremophiles, were roughly distinguished on the basis of their optimal growth conditions [1,2]. Within this frame, halophiles are organisms that require highly saline conditions for survival and proliferation. They inhabit various types of NaCl-saturated environments occurring throughout Earth’s biosphere, spanning a wide range of scales from solar salterns, inland salt-lakes, and deep-sea hypersaline anoxic brines (DHABs) [1,2].

The latter ecosystem is one of the harshest environments on planet Earth and is considered unconducive to the survival of common marine microorganisms. Nevertheless, highly heterogeneous and stratified microbial communities populate the brines and the interfaces of such basins [3].

*Halobacteroides lacunaris* TB21 is an obligate halophilic Gram-negative eubacterium of the order *Halanaerobiales*, able to actively grow in the presence of NaCl concentrations between 1.7 M and 5.5 M. It was recently isolated from the brine of the lately discovered DHAB in the Eastern Mediterranean Sea, *Thetis* [4]. Considered as one of the most saline habitats ever reported to date (350 PSU), *Thetis* is also characterized by anoxia and high pressure (33.5 MPa) conditions. As a consequence, *H. lacunaris* TB21 can be classified as an anaerobic halophilic and barophilic bacterium, namely a polyextremophile. Halophiles and hyperhalophiles are capable, through several strategies, to control the ionic strength inside their cells in order to maintain cellular osmotic pressure. Indeed, it was demonstrated that extremely halophilic obligate anaerobes accumulate high intracellular concentrations of Na^+^ and K^+^ to maintain the cell turgor and to resist the osmotic stress of the hypersaline environment (the so-called “salt inside” strategy) [3,4]. In addition, it was previously demonstrated that barophilic microorganisms have adapted their membrane lipids in order to reach a more fluid architecture thus counteracting the effects of the increase in viscosity due to the high hydrostatic pressure [5,6]. Therefore, these microorganisms have evolved structural and chemical adaptations allowing them to survive and grow in such extreme milieus.

As the majority of Gram-negative bacteria, the external leaflet of the *H. lacunaris* TB21 outer membrane is mainly covered by lipopolysaccharides (LPS) directly interacting with the surrounding environment. Lipopolysaccharides are amphiphilic macromolecules typically built up of three domains: a glycolipid region termed lipid A, embedded in the outer membrane, and a highly variable glycan portion, designated as the O-chain moiety; the bridge between the lipid A and the O-chain is represented by the core oligosaccharide (core OS) [7,8]. The occurrence of the O-chain moiety determines the terminology used to define an LPS molecule: namely, a rough-type LPS (R-LPS) or lipooligosaccharide (LOS) exhibits no O-chain moiety whereas a smooth-type LPS (S-LPS) shows all the above three domains [7,8]. The LPS is widely known to be recognized by the host immune receptorial complex made up of Toll-Like Receptor 4 (TLR4) and myeloid differentiation protein-2 (MD-2), triggering, in a structure-dependent fashion, both the innate and the adaptive immune responses [9,10,11]. Being continuously exposed to environmental stress factors that can likely affect their general structure, LPSs isolated from extremophilic Gram-negative bacteria frequently show unusual chemical features that, as previously mentioned, influence their biological effects on the host immune system. Indeed, it was previously reported that LPSs from non-pathogenic bacteria can act as TLR4/MD-2 antagonists or partial antagonists towards toxic LPSs [12]. As a consequence, there is a constant search for LPS antagonist candidates inspired by a natural source: namely LPS analogues able to bind the receptorial complex, thus competing with toxic LPSs, but not capable of triggering an effective immune response [13].

Given these premises, the establishment of both the structure and the immunological activity of the LPS isolated from a polyextremophile such as *H. lacunaris* TB21 represented an intriguing target to pursue under an evolutionary, chemical, and immunological point of view. Herein we report the structural characterization of the R-LPS from *H. lacunaris* TB21, that turned out to express a unique structure that was established by a combination of organic chemistry, NMR spectroscopy, and mass spectrometry performed on the whole macromolecule and on its fully deacylated product. Furthermore, an accurate investigation of the *H. lacunaris* TB21 R-LPS pro-inflammatory activity on human and murine cell lines and against the toxic LPS from *Escherichia coli* clearly showed its inhibitory action.

## 2. Results

### 2.1. Isolation, Purification, and Compositional Analyses of the R-LPS Isolated from H. lacunaris TB21

The LPS material was extracted from lyophilized bacterial cells by the hot phenol/water procedure [14]. The relative purity and the nature of the extracted sample was evaluated through Sodium Dodecyl Sulphate-PolyAcrylamide Gel Electrophoresis (SDS-PAGE) after silver nitrate gel staining [15] which showed a run to the bottom of the gel typical of a low molecular mass R-type LPS. The extracted R-LPS was subjected to an enzymatic digestion followed by dialysis and size exclusion chromatography to remove cell contaminants. An aliquot of pure R-LPS underwent a detailed monosaccharide and fatty acid compositional investigation.

Monosaccharide analysis revealed the occurrence of D-mannose (D-Man), D-glucose (D-Glc), D-glucuronic acid (D-GlcA), 2-amino-2-deoxy-D-glucose (D-GlcN), L-*glycero*-D-*manno*-heptose (L,D Hep), and 3-deoxy-*manno*-oct-2-ulopyranosonic acid (Kdo). Linkage analysis highlighted the presence of terminal Man, terminal Glc, terminal GlcA, terminal, 3- and 6-substituted GlcN, 2,3-disubstituted Hep, and 5-substituted Kdo.

Investigation of the total fatty acids content on pure R-LPS showed the occurrence of 3-hydroxydodecanoic acid ((*R*)-12:0 (3-OH)), decanoic (10:0), and decenoic acids (10:1); of the latter we have not determined either the position of the double bond or the stereochemistry.

### 2.2. NMR Spectroscopy Structural Characterization of the R-LPS Core OS Isolated from H. lacunaris TB21

In order to determine the structure of the core oligosaccharide region of *H. lacunaris* TB21 R-LPS, a full deacylation, by using anhydrous hydrazine followed by hot strong alkaline treatment [16] on pure R-LPS, was executed to obtain the saccharide fraction (OS) that was then further purified by size-exclusion chromatography. The monosaccharide compositional analysis performed on the OS product confirmed the occurrence of the sugar units found in the pure R-LPS fraction.

The OS product was then subjected to a complete set of homo- and heteronuclear 2D NMR experiments (DQF-COSY, TOCSY, ROESY, NOESY, ^1^H, ^13^C HSQC, ^1^H, ^13^C HMBC and ^1^H, ^31^P HSQC) that allowed the characterization of the whole core OS primary structure. Briefly, each spin system could be assigned by analysis of the DQF-COSY and TOCSY spectra, with the ^1^H, ^13^C HSQC spectrum that led to the identification of each carbon atom. The anomeric configuration of the sugar units was established on the basis of the *intra*-residue NOE contacts and of the ^3^*J*_H-1,H-2_ constant values attained from the DQF-COSY spectrum. In this context, the magnitude of ^3^*J*_H-1,H-2_ with values of 7–9 Hz is associated with the diaxial coupling of a β-configured sugar unit, whereas 2–4 Hz is indicative of an equatorial-axial coupling of α-configured residues. Vicinal ^3^*J*_H,H_ coupling constants allowed the assignment of the relative configuration of each sugar residue. The combination of *inter*-residue NOE effects and long range correlations in the ^1^H, ^13^C HMBC spectrum was fundamental to the establishment of the saccharide domain of the *H. lacunaris* TB21 R-LPS. Finally, ^31^P and ^1^H, ^31^P HSQC experiments were used to establish the location of the phosphate groups decorating the core OS moiety.

**Figure 1:**
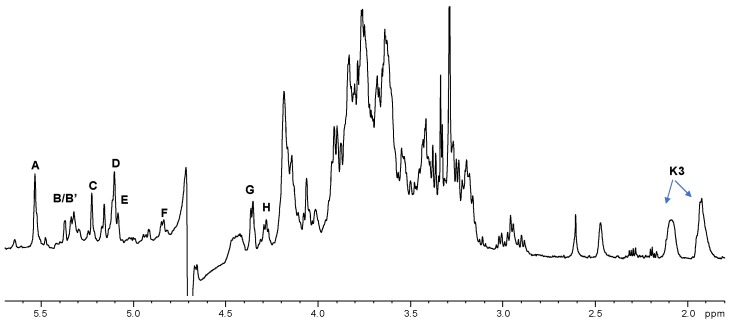
^1^H NMR spectrum of the core oligosaccharide (OS) from *H. lacunaris* TB21 rough-type LPS (R-LPS) obtained after a full deacylation step.

The ^1^H NMR spectrum of the OS product is reported in Figure 1. In the anomeric region of the spectrum, eight different signals were observed as major signals (**A**–**H**, Figure 1, Table 1), whereas at 1.91/2.09 ppm the H-3 methylene proton signals of the Kdo residue (**K**, Figure 1, Table 1) were identified. All the monosaccharide residues were present as pyranose rings, according to both the ^13^C chemical shift values and the presence of long range correlations between C-1/H-1 and H-5/C-5 in the ^1^H, ^13^C HMBC spectrum (for the Kdo residue between C-2 and H-6) [17,18,19,20]. The ^31^P and ^1^H, ^31^P HSQC experiments (not shown) revealed three different monophosphate ester groups with chemical shifts between −0.70 ppm and 3.55 ppm (Table 1), all of which were correlated with sugar proton signals.

**Figure 2:**
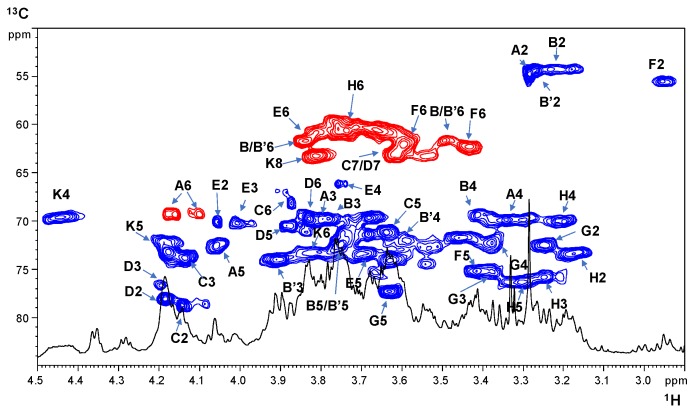
Zoom of the ^1^H, ^13^C Heteronuclear Single Quantum Coherence spectroscopy (HSQC) spectrum of the core OS product obtained after a full deacylation step of *H. lacunaris* TB21 R-LPS. Most of the hetero-nuclear correlations are indicated. The color of the cross peaks indicates the phase: blue indicates CH and CH_3_ signals whereas red indicates CH_2_ signals.

**Figure 3:**
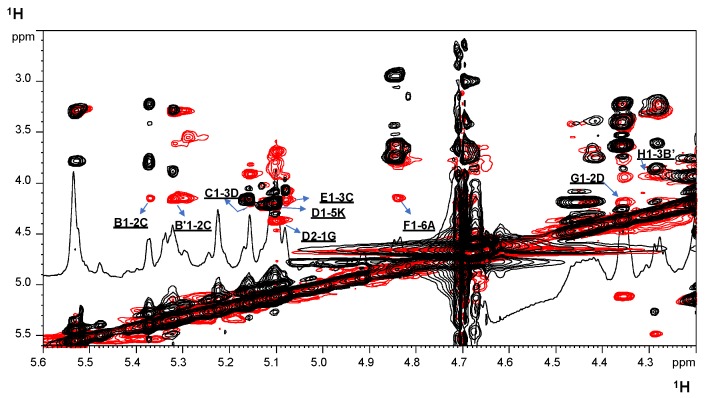
Zoom of the overlapped NOESY (black) and TOCSY (red) spectra of the core OS product obtained after a full deacylation step of *H. lacunaris* TB21 R- LPS. The key *inter*-residue NOE contacts are indicated.

Spin systems **A** and **F** (H-1 at *δ* 5.52 and 4.83 ppm, respectively, Table 1) were assigned to α-GlcN and β-GlcN of the lipid A domain based on their H-2 proton signals correlating with two nitrogen-bearing carbon atoms at *δ* 54.7 and 55.6 ppm (Figure 2), respectively. Their *gluco* configuration was indicated by the high ^3^*J*_H,H_ ring proton values (8–10 Hz). The observation of the *inter*-residue NOE correlation between H-1 of **F** and H-6 of **A** (*δ* 4.17 ppm, Table 1), attained from the NOESY spectrum (Figure 3), validated the assignment of residues **A** and **F** to the lipid A disaccharide backbone. Both GlcN units were phosphorylated, as proven by the observation of downfield shifts for C-1/H-1 **A** (*δ* 90.4/5.52 ppm) and C-4/H-4 **F** (*δ* 74.0/3.63 ppm) in addition to the occurrence of correlations in the ^1^H, ^31^P HSQC spectrum (not shown), with the signal at *δ* 3.55 ppm for residue **A** and at *δ* 2.02 ppm for residue **F**.

The spin systems **C** (H-1 at *δ* 5.15 ppm) and **D** (H-1 at *δ* 5.10 ppm) were identified as α-*manno* configured residues due to the low coupling constant values ^3^*J*_H1,H2_ and ^3^*J*_H2,H3_ (below 3 Hz), indicative of the H-2 equatorial orientation; furthermore, in the TOCSY spectrum, it was possible to assign from H-2 all the other ring proton resonances allowing their assignment as heptose units. The chemical shift value of C-6 of **C** and **D** (Figure 2, Table 1) led to their final identification as L-*glycero*-D-*manno*-heptose residues, in agreement with the compositional analysis. Spin systems **B** (H-1 at *δ* 5.37 ppm) and **B’** (H-1 at *δ* 5.31 ppm), assigned to *gluco*-configured residues, possessed a signal for C-2 at *δ* 54.3 ppm (Figure 2, Table 1) indicating that C-2 of both spin systems was a nitrogen-bearing carbon atom; thus, spin systems **B** and **B’** were identified as α-GlcN residues. Spin system **E** (H-1 at *δ* 5.07 ppm) was identified as an α-Man, as proven by the ^3^*J*_H,H_ coupling constant values, whereas the α-anomeric configuration was assigned, as stated above, on the basis of the ^1^*J*_C,H_ values and the *intra*-residue NOE connectivity of H-1 only with H-2. Spin system **G** (H-1 at *δ* 4.35 ppm) was assigned to a β-GlcA unit as proven by its C-6 occurring at 175.8 ppm, its large ^3^*J*_H-1,H-2_ value and the presence of NOE correlations between the anomeric proton signal and both H-3 and H-5 signals. Finally, spin system **H** (H-1 at *δ* 4.28 ppm) was attributed to a β-Glc unit. The identification of the Kdo (**K**) was achieved starting from the diastereotopic methylene signal (*δ* 1.91/2.09 ppm, Figure 1 and Table 1), and its anomeric α-configuration was assigned on the basis of the chemical shift values of H-3 and of the ^3^*J*_H-7,Ha-8_ and ^3^*J*_H-7,Hb-8_ coupling constants [18,19,20,21].

Low-field shifted carbon signals, observed in the ^1^H, ^13^C HSQC spectrum (Figure 2), were pivotal to identify substitutions at O-2 and O-3 of residue **C**, O-2 and O-3 of **D**, O-3 of **B’**, O-6 of **A** and **F**, and O-5 of **K**; whereas **B**, **E**, **G**, and **H** were identified as terminal sugar units.

By merging the data from the NOESY, ROESY, and ^1^H, ^13^C HMBC spectra, it was possible to define the primary sequence of the *H. lacunaris* TB21 R-LPS core OS. In detail, starting from the lipid A disaccharide backbone composed of residues **A** and **F**, this latter was found to be substituted at position O-6 by the Kdo residue (**K**). This was also confirmed by the observation of the weak downfield shift of signal of C-6 of residue **F**, which is consistent with the expected α-(2→6) ketosidic linkage of Kdo with the β-GlcN unit [22,23]. The Kdo residue was, in turn, substituted at O-5 by L,D-heptose **D**, as proven by the NOE correlation observed between the anomeric proton signal of **D** (δ 5.10 ppm) and H-5 signal of **K** (*δ* 4.18 ppm) (Figure 3); this was further confirmed by the observation of the respective long range correlation in the ^1^H, ^13^C HMBC spectrum (not shown). Heptose **D** was, in turn, substituted by heptose **C** at its O-3 position and by β-GlcA **G** unit at its O-2 position, as suggested by the NOE contacts found between H-1 **G** (*δ* 4.35 ppm) and H-2 **D** (*δ* 4.18 ppm) and between H-1 **C** (*δ* 5.15 ppm) with H-3 of **D** (*δ* 4.19 ppm) (Figure 3).

The GlcN **B**/**B’** unit was found linked to position O-2 of heptose **C**, as proven by the NOE correlation between the anomeric proton signal of **B**/**B’** (*δ* 5.37/5.31 ppm) and H-2 of **C** (*δ* 4.14 ppm) (Figure 3). The heptose **C** was also substituted, at its O-3 position, by α-Man **E** as attested by the NOE contact visible between H-1 of **E** (*δ* 5.07 ppm) and H-3 of **C** (*δ* 4.13 ppm) (Figure 3). Finally, the observation of a NOE correlation between H-1 of β-Glc unit **H** and H-3 of residue **B’** (*δ* 3.90 ppm) (Figure 3), in addition to the respective HMBC correlation, allowed us to define the linkage **H**1→3**B’**.

**Scheme 1:**
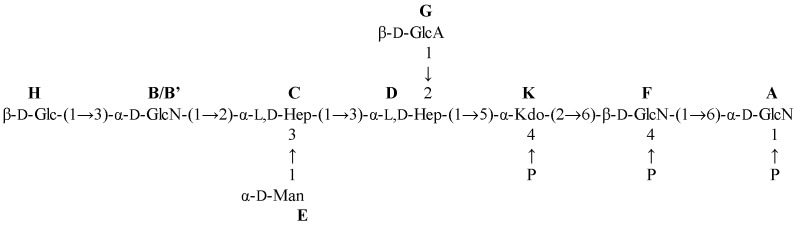
Core OS structure of the R-LPS from *H. lacunaris* TB21.

Together, methylation analysis and NMR spectroscopy led to establishing the complete OS sequence from the *H. lacunaris* TB21 R-LPS, as a heptasaccharide containing a terminal β-Glc unit in a non-stoichiometric fashion. The elucidated core OS structure is depicted in Scheme 1 and Figure S1.

### 2.3. MALDI Mass Spectrometry Investigation of the Lipid A Isolated from H. lacunaris TB21 R-LPS.

In order to unveil the complete structure of the glycolipid portion of the R-LPS from *H. lacunaris* TB21, an aliquot of the pure R-LPS underwent a mild acid hydrolysis to cleave the labile glycosidic linkage between the Kdo and the non-reducing glucosamine of the lipid A disaccharide backbone. 

**Figure 4:**
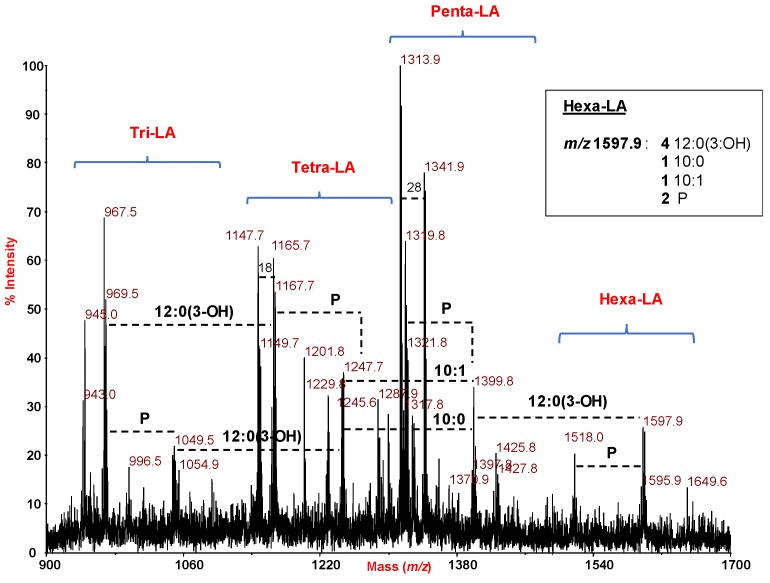
Reflectron Matrix-Assisted Laser Desorption Ionization Time of Flight (MALDI-TOF) mass spectrum of the lipid A from *H. lacunaris* R-LPS. Only deprotonated ions [M − H]^−^ are formed in these conditions. The lipid A species are outlined. The structural composition of the ion at *m/z* 1597.9 is listed to the right.

MALDI MS and MS/MS investigation on isolated lipid A was executed. The reflectron MALDI mass spectrum, recorded in negative polarity, is reported in Figure 4. It clearly showed the occurrence of a mixture of lipid A species differing in the pattern of fatty acids and in phosphate content. In detail and on the basis of fatty acid analysis, the ion at *m/z* 1597.9 (Figure 4) was attributed to a *bis*-phosphorylated hexa-acylated lipid A species carrying four units of 12:0 (3-OH), one 10:0 and one 10:1. The relative *mono*-phosphorylated lipid A form was also assignable to the species at *m/z* 1518.0 (Figure 4). A *bis*-phosphorylated penta-acylated lipid A species lacking one 12:0 (3-OH) residue was attributed to the peak at *m/z* 1399.8, whose *mono*-phosphorylated form could be assigned to the peak at *m/z* 1319.8. The mass range *m/z* 1147.7–1247.7 showed peaks assignable to tetra-acylated lipid A species; as an example, the ion peak at *m/z* 1167.7 was attributed to a *mono*-phosphorylated lipid A species lacking one 12:0 (3-OH) and one 10:1 unit, whereas the ion peak at *m/z* 1245.6 was assigned to a *bis*-phosphorylated form lacking one 10:0 and one 12:0 (3-OH) residue (Figure 4).

**Figure 5:**
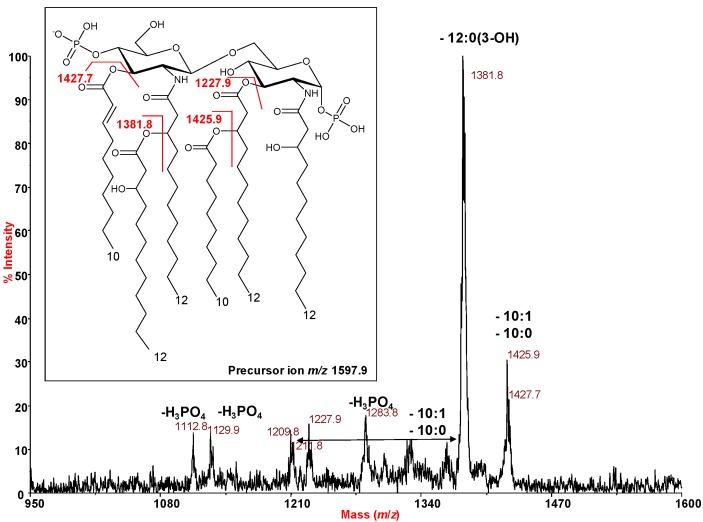
MALDI tandem mass spectrometry (MS/MS) analysis of the hexa-acylated lipid A species at *m/z* 1597.9 from *H. lacunaris* TB21 R-LPS. Fragment assignments are indicated. The double bond position is tentative and remains to be defined.

A detailed negative-ion MS/MS analysis was conducted to reveal the location of the lipid A acyl moieties with respect to the glucosamine backbone. In detail, the MS/MS spectrum of the precursor ion at *m/z* 1597.9 (Figure 5) showed a main product ion at *m/z* 1381.8 matching with a lipid A fragment lacking one 12:0 (3-OH) unit. Further product ions were also detected at *m/z* 1425.9 and 1427.7 indicative of one 10:0 and one 10:1 fatty acid loss, respectively. Similarly, the MS/MS spectrum of the precursor ion at *m/z* 1399.7 (Figure S2), relative to a *bis*-phosphorylated penta-acylated lipid A species, revealed product ions at *m/z* 1227.8 and 1229.7 matching with the lipid A fragments originated from one 10:0 and one 10:1 fatty acid loss, respectively.

More interestingly, a product ion at *m/z* 1029.5 was identified as a fragment lacking both 12:0 (3-OH) and 10:0 fatty acids. MS/MS analysis executed on the precursor ion at *m/z* 1245.6 (Figure S3), relative to a *bis*-phosphorylated tetra-acylated lipid A species, allowed for the location of the primary acyl chains with respect to the disaccharide backbone. Briefly, the negative-ion MS/MS spectrum highlighted, besides product ions arising from the lipid A fragments lacking one 12:0 (3-OH) fatty acid (*m/z* 1029.6) or one 10:1 fatty acid (*m/z* 1075.6), also an important peak originating from the cleavage of the glycosidic linkage (Y_1_) [24] at *m/z* 654.4 (Figure S3). This product ion allowed the definition of the acyl chain moieties decorating the reducing glucosamine unit, namely two 12:0 (3-OH) residues, thus suggesting the occurrence, on the non-reducing glucosamine, of one 12:0 (3-OH) and one 10:1 moieties. A further confirmation of such a structure was furnished by the negative-ion MS/MS analysis executed on the precursor ion at *m/z* 1047.5 (Figure S4), relative to a *bis*-phosphorylated tri-acylated lipid A species carrying two 12:0 (3-OH) in amide-linkage and one 10:1 as primary ester-linked fatty acid of the non-reducing glucosamine unit. This was proven by the presence, in the MS/MS spectrum, of the Y_1_ ion at *m/z* 456.1 and of a product ion at *m/z* 877.4 assigned to a lipid A fragment originated from the loss of a 10:1 fatty acid (Figure S4).

### 2.4. Immunological Properties of Isolated H. lacunaris TB21 R-LPS

We evaluated the impact of *H. lacunaris* TB21 R-LPS on the human innate immune system in vitro. First, in order to confirm the absence of contamination, the pure R-LPS was tested for the presence of bacterial lipoproteins (BLP) in HEK 293 cells expressing TLR2 [25], which is the receptor of BLP (Figure S4). Both the NF-κB activation and IL-8 release represented the read out of the experiment. No activation of NF-κB as well as no release of IL-8 were recorded (Figure S5). In view of this, the immunological properties of *H. lacunaris* TB21 R-LPS were assessed in the model of HEK293 cells stably expressing human CD14, MD-2, and human TLR4. HEK 293 hTLR4 cells were exposed to different *H. lacunaris* TB21 R-LPS concentrations (namely 1, 10, and 100 ng/mL). The hexa-acylated potent agonistic LPS from *E. coli* was used as a positive control at the same concentrations as above. NF-κB activation was evaluated through the assessment of luciferase activity after 4 h stimulation; whereas IL-8 release was recorded via enzyme-linked immunosorbent assay (ELISA) after 18 h stimulation.

**Figure 6:**
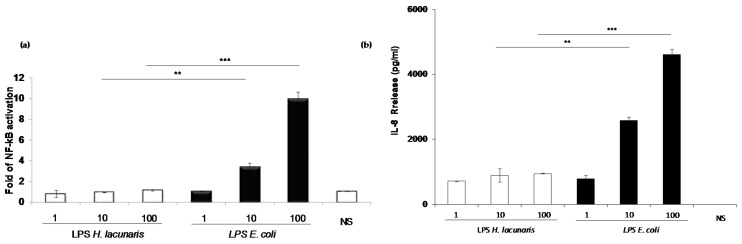
Stimulation of HEK 293-hTLR4/MD-2/CD14 with R-LPS derived from *H. lacunaris* TB21. Fold of activation of NF-κB (**a**) and IL-8 (**b**) release after 4 h and 18 h, respectively, of stimulation with 1, 10, and 100 ng/mL of R-LPS; commercial LPS of *E. coli* was used as a control. Significant difference between *H. lacunaris* TB21 R-LPS generated values and the corresponding *E. coli* LPS values are indicated (*H. lacunaris* TB21 R-LPS vs. *E. coli* LPS) (** *p* < 0.01; *** *p* < 0.001).

The luciferase assay results highlighted that the *H. lacunaris* TB21 R-LPS induced a significantly lower NF-κB activation with respect to cells treated with *E. coli* LPS (R-LPS of *H. lacunaris* TB21 vs. LPS of *E. coli p* < 0.01 at 10 ng/mL and *p* < 0.001 at 100 ng/mL) (Figure 6a). In agreement with these results, the level of IL-8 release was lower after stimulation with *H. lacunaris* TB21 R-LPS with respect to LPS from *E. coli* LPS (*H. lacunaris* TB21 R-LPS vs. *E. coli* LPS *p* < 0.01 at 10 ng/mL and *p* < 0.001 at 100 ng/mL) (Figure 6b).

Then, we expanded the assessment of the immunopotential of *H. lacunaris* TB21 R-LPS through its analysis in mouse bone marrow derived macrophages (BMDMs), which are a cell population naturally committed to sense bacterial PAMPs (Pathogen Associated Molecular Patterns). BMDMs were exposed to different *H. lacunaris* TB21 R-LPS concentrations (1, 10, and 100 ng/mL) for 6 h. The LPS from *E. coli* was used as a positive control in parallel, as above. The measurement of the release of relevant inflammatory cytokines, i.e., Tumor necrosis factor-α (TNF-α), chemokine (KC) (IL-8), Interleukin-6 (IL-6), and C-C Motif Chemokine Ligand 5 (CCL-5) was the read-out of this experiment. As shown in Figure S6, the *H. lacunaris* TB21 R-LPS produced minimal release of these cytokines thereby confirming that it was unable to elicit an inflammatory response.

**Figure 7:**
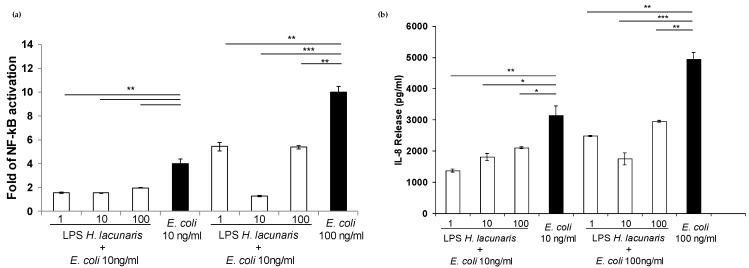
Competition assay. (**a**) Fold of NF-κB activation and (**b**) IL-8 release in HEK 293 hTLR4/MD-2/CD14 stimulated with different concentrations (1, 10, and 100 ng/mL) of *H. lacunaris* TB21 R-LPS and then re-stimulated with 10 ng/mL and 100 ng/mL of *E. coli* LPS (LPS-EB ultrapure, InvivoGen). Significant difference between *H. lacunaris* TB21 R-LPS generated values and the corresponding *E. coli* LPS values are indicated (*H. lacunaris* TB21 R-LPS vs. *E. coli* LPS) (* *p* < 0.05; ** *p* < 0.01; *** *p* < 0.001).

We then proceeded to evaluate the capability of *H. lacunaris* TB21 R-LPS to interfere with the TLR4-mediated signaling induced by *E. coli* LPS. In order to assess this property, HEK 293 hTLR4 cells were pre-incubated for 1 h with different amounts (1, 10, and 100 ng/mL) of *H. lacunaris* TB21 R-LPS and then exposed to 10 ng/mL of *E. coli* LPS for 4 h. *H. lacunaris* TB21 R-LPS significantly antagonized *E. coli* LPS-dependent TLR4-mediated NF-κB activation as well as IL-8 expression at all the concentrations tested (NF-κB activation, *H. lacunaris* TB21 R-LPS vs. *E. coli* LPS *p* < 0.01 at 1, 10, and 100 ng/mL; IL-8 release, *H. lacunaris* TB21 R-LPS vs. *E. coli* LPS *p* < 0.01 at 1 ng/mL and *p* < 0.05 at 10 and 100 ng/mL) (Figure 7a,b). Intriguingly, when HEK 293 hTLR4 cells were pre-incubated with *H. lacunaris* TB21 R-LPS and then exposed to 100 ng/mL of *E. coli* LPS, the inhibitory activity of the polyextremophile R-LPS resulted in higher measurements for both the NF-κB and IL-8 levels (NF-κB activation, *H. lacunaris* TB21 R-LPS vs. *E. coli* LPS *p* < 0.01 at 1 and 100 ng/mL and *p* < 0.001 at 10 ng/mL; IL-8 release, *H. lacunaris* TB21 R-LPS vs. *E. coli* LPS *p* < 0.01 at 1 and 100 ng/mL and *p* < 0.001 at 10 ng/mL) (Figure 7a,b).

The combination of these data led to the conclusion that *H. lacunaris* TB21 R-LPS exerts a very low immunostimulant activity on HEK 293 hTLR4 cells. More interestingly, it additionally exerts a good TLR4/MD-2 antagonistic activity towards the potent agonist *E. coli* LPS at all the concentrations used in the assays.

## 3. Discussion

In this paper the characterization of the full structure of the R-LPS isolated from the polyextremophilic bacterium *H. lacunaris* TB21, is reported. Briefly, the core OS moiety turned out to be a *mono*-phosphorylated heptasaccharide containing, among others, heptoses and glucuronic acid. The lipid A was revealed to be composed of a glucosamine disaccharide backbone decorated by one or two phosphate groups and acylated by (*R*)-12:0 (3-OH) in amide linkage, (*R*)-12:0 (3-OH) and 10:1 as primary ester-linked fatty acids, and (*R*)-12:0 (3-OH) as a secondary acyl chain.

The structural investigation highlighted an R-LPS which was highly negatively charged at physiological pH since it exhibits three phosphate groups and two sugar units bearing carboxyl groups (namely, the Kdo and the glucuronic acid residues). The occurrence of various decorations by anionic substituents on the R-LPS may be designed as an important evolutionary adaptation since such negative charges could act as a buffering system, able to regulate the pH on the external membrane surface, thus protecting the bacterium from the extreme salinity conditions. Furthermore, the presence of short, as well as, unsaturated acyl chains composing the lipid A moiety can be considered a direct consequence of the necessity of *H. lacunaris* TB21 to reach a good outer membrane fluidity, thus protecting the entire bacterial cell from the high pressure conditions of the DHAB *Thetis* [14].

It is widely known that LPS is involved in innate immune system elicitation where the hexa-acylated *bis*-phosphorylated lipid A from *E. coli* exhibits a high immunostimulant activity binding to TLR4/MD-2 complex and triggering the downstream signaling [9,10,11]. It is established that subtle chemical modifications in certain parts of the LPS structure are responsible for fine-tuning of the innate immunity response [9,10,11,26]. The search for LPS analogues, isolated from a natural source, that can act as antagonists on TLR4/MD-2 is a hot topic with a growing interest in many research fields. Indeed, this paves the way to the possibility to develop ad-hoc synthesized compounds able to limit the lethal consequences due to the excessive and deregulated TLR4 activation and signaling. This fascinating opportunity prompted us to investigate the immunostimulatory activity of *H. lacunaris* TB21 R-LPS which revealed a very weak ability to engage the TLR4/MD-2/CD14 pathway compared to the LPS of *E. coli*. This represents an intriguing discovery since *H. lacunaris* TB21 R-LPS was characterized by a mixture of tetra-, penta-, and hexa-acylated lipid A species with this latter form considered, as stated above, the prototype of the agonistic activity on the TLR4/MD-2 complex. Nevertheless, several structural differences may be at the basis of the divergent immunological activity exerted by *E. coli* LPS and *H. lacunaris* TB21 R-LPS, namely (i) the symmetric distribution of the acyl moieties of *H. lacunaris* TB21 lipid A which is characterized, in its hexa-acylated form, by three fatty acids on each glucosamine unit; (ii) the presence in the polyextremophile lipid A of acyl chains shorter than those of *E. coli* (10 and 12 carbon atoms vs. 14 and 12 carbon atoms) as well as the occurrence of unsaturated acyl moieties (namely 10:1). More intriguingly, the *H. lacunaris* TB21 R-LPS exhibited a potent inhibitory activity towards the toxic effects of *E. coli* LPS on HEK 293 hTLR4 cells. This interesting property has been extensively reported for tetra-acylated lipid A, such as lipid IV_A_ [27], and also for some other extremophilic LPSs, such as those isolated from the thermophile *Thermomonas hydrothermalis* [28] and the halophile *Halomonas megadiensis* [29]. The TLR4/MD-2 antagonistic activity of *H. lacunaris* TB21 R-LPS may be explained with the occurrence of hypo-acylated lipid A species, namely tetra- and penta-forms, in addition to the hexa-acylated forms, thus resulting in a very weak elicitation of an immune response but with a fascinating capability to robustly compete with the toxic *E. coli* LPS for binding to the TLR4/MD-2 complex.

Therefore, besides the peculiar structural properties observed for *H. lacunaris* TB21 R-LPS, this work clearly demonstrates that the lipid A isolated from this polyextremophilic bacterium represents a further attractive example of the potential use of extremophiles in the research of LPS-analogues that can act as immunomodulators of the immune response [14].

## 4. Materials and Methods

### 4.1. Bacteria Isolation and Growth

Brine samples (salinity, 340 g/kg) were obtained from deep-sea hypersaline anoxic lake *Thetis* (34.6698° N and 22.1455° E) from the depth of 3300 m. Sampling was done during MICRODEEP12 cruise (September 2012) on board the RV *Urania*. For cultivation, 50 mL of brine was mixed with sterile mineral base medium containing (g/L): 240 NaCl, 5 KCl, 2 K_2_HPO_4_, and 0.5 NH_4_Cl. The mineral base medium was supplemented with yeast extract (40 mg/L), selenite/tungstate (20 mg/L), trace elements, and vitamins solution (1 mL/L each) [30]. The medium was previously adjusted to pH 7.0 with 50 mM of HEPES and 1.0 M NaOH, boiled under N_2_-CO_2_ atmosphere (80:20 *v/v*), dispensed into 120 mL serum bottles under N_2_:CO_2_ (80:20 *v/v*) and autoclaved. After sterilization, 0.5 g/L filter sterilized Na_2_S and 15 mM of glycine betaine (GB) were added. Cultures were incubated at 20 °C temperature in the dark for 2 months. Positive enrichments were transferred into fresh medium (salinity, 240 g/L) supplemented with 15 mM of GB and used for further isolation and phylogenetic analysis. Hypersaline (salinity, 320 g/L) DSMZ 764 medium [30] was used for the isolation of bacterial members of the *Thetis* enrichment. Pure cultures were obtained by serial dilutions to extinction and the final isolates were checked microscopically and by 16S rRNA sequencing. Colony formation was possible only in Hungate roll tubes or on plates incubated in anaerobic jars. The colony transfer was repeated at least twice before the cultures were considered pure.

To obtain a sufficient amount of biomass for R-LPS isolation and characterization, growth of polyextremophilic bacterium *H. lacunaris* TB21 was performed at 37 °C in 120 mL serum bottles with butyl rubber stoppers filled to 90% with liquid DSMZ 764 medium. The medium was made anoxic first by “cold boiling” upon evacuation followed by 3 cycles of flushing with argon-evacuation. Anaerobic conditions were achieved by the final addition of 0.2 mM H_2_S. The cultures were incubated for 10 days with periodic shaking of the flasks.

### 4.2. Isolation and Purification of the R-LPS from H. lacunaris TB21

Lyophilized bacterial cells were extracted following the hot phenol/water protocol [14]. The extracted material was exhaustively dialyzed against distilled water. In order to define the nature of the extracted material, an SDS-PAGE followed by silver nitrate gel staining [15] was executed revealing the rough nature of the isolated LPS. To remove cell contaminants, such as proteins and nucleic acids, an enzymatic digestion with RNase (Sigma-Aldrich, Darmstadt, Germany), DNase (Sigma-Aldrich, Darmstadt, Germany), and Proteinase K (Sigma-Aldrich, Darmstadt, Germany) (37 °C and 56 °C) was performed. To further purify the R-LPS material, an ultracentrifugation step (4 °C, 100,000 *g*, 16 h) and a size-exclusion chromatography on a Sephacryl High Resolution S-200 (GE Healthcare, Little Chalfont, UK) column were executed.

### 4.3. Chemical Analyses

The monosaccharide content, both on intact R-LPS and the isolated core OS product, was determined by analysis of the acetylated *O*-methyl glycoside derivatives obtained by treatment with HCl/MeOH (1.25 M, 85 °C) followed by an acetylation step with acetic anhydride in pyridine (85 °C, 30 min). The absolute configuration was defined through the evaluation of the *O*-octylglycoside derivatives as previously described [31]. To establish the sugar linkage pattern, an aliquot of R-LPS was methylated with CH_3_I, hydrolyzed with trifluoroacetic acid (4 M, 100 °C, 4 h), carbonyl reduced with NaBD_4_, and acetylated [32,33].

The total fatty acid content was determined on intact R-LPS by treating with HCl (4 M, 100 °C, 4 h) followed by NaOH (5 M, 100 °C, 30 min). The pH was adjusted to 3. An extraction in chloroform allowed us to isolate the fatty acids that were then methylated with diazomethane. The absolute configuration of acyl chains was determined as previously described [34]. Authentic 3-hydroxy fatty acids were used to assign the (*R*) configuration to the R-LPS fatty acids.

All chemical analyses were performed by means of gas-liquid chromatography (GLC-MS) Agilent Technologies 6850A (Santa Clara, CA, USA) equipped with a mass selective detector 5973N and a Zebron ZB-5 capillary column (Phenomenex, 30 m × 0.25 mm i.d., 0.25 μm as film thickness, flow rate 1 mL/min, He as carrier gas) and using the temperature program 140 °C for 3 min, 140 °C/240 °C at 3 °C/min.

### 4.4. Isolation of the Core OS from the H. lacunaris TB21 R-LPS

An aliquot of pure R-LPS was treated with anhydrous hydrazine under magnetic stirring (37 °C, 90 min), cooled, poured into ice-cold acetone, and allowed to precipitate. The precipitate was then centrifuged (3000 *g*, 30 min), washed with ice-cold acetone, dried, dissolved in water, and lyophilized. The *O*-deacylated product was then *N*-deacylated with KOH (4 M, 120 °C). A gel filtration chromatography was performed to remove salts [16]. To further purify the core OS product, a further gel filtration on a BioRad Bio-Gel P4 Fine packed column was executed. Gel filtration chromatography was run at room temperature with a flow rate of 8 cm/hr, using distilled and degassed water as eluent. The column was calibrated using Bio-Rad’s Gel Filtration Standards.

### 4.5. NMR Spectroscopy

1D and 2D ^1^H NMR spectra were performed at 298 K in D_2_O at pD = 7 with a Bruker 600 DRX spectrometer (Billerica, MA, USA) equipped with a cryoprobe. The spectra were calibrated with internal acetone (δ_H_ = 2.225 ppm; δ_C_ = 31.45 ppm). ^31^P NMR experiments were carried out with a Bruker DRX-400 spectrometer; aqueous 85% phosphoric acid was used as external reference (δ = 0.00 ppm). Total correlation spectroscopy (TOCSY) experiments were performed with spinlock times of 100 ms by using data sets (*t*1 × *t*2) of 4096 × 512 points. Rotating frame Overhauser enhancement spectroscopy (ROESY) and Nuclear Overhauser enhancement spectroscopy (NOESY) experiments were executed by using data sets (*t*1 × *t*2) of 4096 × 512 points with mixing times between 100 and 300 ms. Double-quantum-filtered phase sensitive correlation spectroscopy (DQF-COSY) experiments were executed by using data sets of 4096 × 900 points. The data matrix in all the homonuclear experiments was zero-filled in both dimensions to give a matrix of 4K × 2K points and was resolution-enhanced in both dimensions by a cosine-bell function before Fourier transformation. Coupling constants were determined by 2D phase-sensitive DQF-COSY [35,36].

Heteronuclear single quantum coherence (HSQC) and heteronuclear multiple bond correlation (HMBC) experiments were executed in ^1^H-detection mode by single-quantum coherence with proton decoupling in the ^13^C domain using data sets of 2048 × 256 points. HSQC was performed using sensitivity improvement and in the phase-sensitive mode using Echo/Antiecho gradient selection, with multiplicity editing during the selection step [37]. HMBC was optimized on long range coupling constants, with a low-pass *J*-filter to suppress one-bond correlations, using gradient pulses for selection, and a 60 ms delay was used for the evolution of long-range correlations. ^1^H, ^31^P HSQC was optimized for an 8 Hz coupling constant. The data matrix in all the heteronuclear experiments was extended to 2048 × 1024 points by using forward linear prediction extrapolation [38].

### 4.6. MALDI Mass Spectrometry

MALDI TOF MS of the lipid A fraction, recorded in reflectron mode and negative polarity, was executed on a 4800 Proteomic analyzer (SCIEX, Concord, ON, Canada) supplied with a Nd: YAG laser (wavelength of 355 nm). Lipid A preparation was performed as previously described [17,18]. Briefly, the sample was dissolved in CHCl_3_/CH_3_OH (50:50 *v/v*) at a concentration of 25 pmol/μL. The matrix solution was trihydroxyacetophenone (THAP) dissolved in CH_3_OH/0.1% TFA/CH_3_CN (7:2:1 *v/v/v*) at a concentration of 75 mg/mL. A sample/matrix solution mixture (1:1 *v/v*) was deposited (1 μL) onto a stainless steel gold-plated 100-sample MALDI probe tip, and left to dry at room temperature. Mass accuracy was found below 75 ppm. 2000 laser shots were approximately accumulated for each mass spectrum, whereas 4000/6000 shots were summed for MS/MS acquisitions. A series of MALDI TOF/TOF experiments were performed with helium, argon, xenon or air as the collision gas, and the resulting fragmentation spectra were compared with those obtained without collision induced dissociation (CID); no significant differences were observed in all the observed cases in the mass range above *m/z* 200. Below this mass, product ions corresponding to the phosphate fragments at *m/z* 79 and 97 were much more abundant in the CID spectra regardless of the collision gas.

### 4.7. Cell Cultures

The stably transfected human embryonic kidney epithelial cell line HEK 293-hTLR4/MD2-CD14 and HEK293 hTLR2 (InvivoGen, San Diego, CA, USA) were cultured in DMEM with 10% FBS (Eurclone, Milan, Italy), 10 μg/mL Blasticidin-S and 50 μg/mL HygroGold^®^ (both by InvivoGen, San Diego, CA, USA).

### 4.8. BMDMs Isolation and Culture

C57BL/6 mice were purchased from Charles River (Charles River ITALY). BMDMs were derived from the bone marrow cells collected from five-week old female mice, as already reported [39]. Subsequently, BMDMs were differentiated during 5 days in RPMI1640 (Lonza, Basel, Switzerland), supplemented with 10% FBS (Hyclone, San Angelo, TX, USA), 1% Glutamine (Lonza, Basel, Switzerland), 1% Na pyruvate (Lonza, Basel, Switzerland), 1% NEAA (Lonza, Basel, Switzerland), 0.5% 2-ME (Thermo Fisher Scientific, Milan,, Italy), and 30 ng/mL macrophage colony-stimulating factor (M-CSF; Miltenyi Biotec, Bergisch Gladbach, Germany).

### 4.9. Cytokine Measurement

Human cytokine IL-8 was determined in the supernatants of stimulated HEK 293-TLR4/MD2-CD14 and HEK 293-TLR2 cells by using R&D Systems DuoSet ELISA (R&D Systems, Minneapolis, MN, USA). Murine cytokines were determined in supernatants of stimulated BMDMs by using R&D Systems DuoSet ELISA (R&D Systems, Minneapolis, MN, USA) kits for TNF-α, IL-6, KC, and CCL-5 according to the manufacturers’ instructions.

### 4.10. HEK 293 hTLR4/CD14/MD2 Cell Culture, Transfection, and Stimulation

Stably transfected cell line HEK 293-TLR4/MD2-CD14 or HEK 293 hTLR2 were seeded into 96-well plates at the concentration of 3 × 105 cells/mL. The next day cells were transiently transfected with PolyFect Transfection Reagent (Qiagen, Hilden, Germany) with a reaction mix composed by 150 ng of Firefly luciferase reporter constructs, pGL3.ELAM.tk (containing NF-κB promoter sequences), and 15 ng of *Renilla* luciferase reporter plasmid, pRLTK (as an internal control). Twenty-four hours post-transfection cells were untreated or incubated with different concentrations (1, 10, and 100 ng/mL) of R-LPS of *H. lacunaris* TB21 or of purified *E. coli* LPS (LPS-EB ultrapure; InvivoGen, San Diego, California, USA) for 4 h to analyze the NF-κB activity or for 18 h to measure the IL-8 release, respectively. For the competition assays, HEK 293-TLR4/MD2-CD14 cells were primed with LPS (1, 10 and 100 ng/mL) of R-LPS of *H. lacunaris* TB21 for 1 h and then stimulated with 10 or 100 ng/mL of *E. coli* LPS for 4 h. After this time, the NF-κB activity and IL-8 release were measured.

HEK 293 hTLR2 were exposed to R-LPS of *H. lacunaris* TB21 as above and Pam3CSK4 (1 µg/mL, InvivoGen, San Diego, CA, USA) was used as the positive control. After 6 h, NF-κB activity was measured and IL-8 release was recorded via ELISA assay after 18 h of stimulation.

## Figures and Tables

**Table 1:** ^1^H, ^13^C (*italic*), and ^31^P (**bold**) chemical shifts (ppm) of deacylated R-LPS from *H. lacunaris* TB21.

Unit	1	2	3	4	5	6	7	8
A 6-α-GlcN1*P*	5.52	3.28	3.79	3.34	4.06	4.17/4.10	-	-
	*90.4*	*54.7*	*69.8*	*69.3*	*72.7*	*69.6*	-	-
	3.55	-	-	-	-	-	-	-
B *t*-α-GlcN	5.37	3.23	3.77	3.41	3.77	3.84/3.48	-	-
	*94.7*	*54.3*	*69.8*	*69.3*	*72.9*	*62.7*	-	-
B’ 3-α-GlcN	5.31	3.25	3.90	3.58	3.76	3.84/3.48	-	-
	*98.0*	*54.3*	*74.0*	*71.9*	*73.0*	*62.7*	-	-
C 2,3-α-Hep	5.15	4.14	4.13	3.71	3.63	3.87	3.61	-
	*100.8*	*78.7*	*74.0*	*71.3*	*71.2*	*68.4*	*63.0*	-
D 2,3-α-Hep	5.10	4.18	4.19	3.73	3.88	3.83	3.62	-
	*98.0*	*78.1*	*76.8*	*71.2*	*70.5*	*69.7*	*63.0*	-
E *t*-α-Man	5.07	4.05	4.00	3.75	3.70	3.81/3.61	-	-
	*102.2*	*70.1*	*70.3*	*66.2*	*73.3*	*61.0*	-	-
F 6-β-GlcN4*P*	4.83	2.95	3.75	3.63	3.43	3.60/3.43	-	-
	*99.3*	*55.6*	*72.8*	*74.0*	*75.2*	*62.3*	-	-
	-	-	-	2.02	-	-	-	-
G *t*-β-GlcA	4.35	3.24	3.40	3.38	3.63	-	-	-
	*102.3*	*72.4*	*75.2*	*72.4*	*77.3*	*175.8*	-	-
H *t*-β-Glc	4.28	3.16	3.24	3.20	3.32	3.75/3.66	-	-
	*102.8*	*73.2*	*75.8*	*69.8*	*76.2*	*60.6*	-	-
K 5-α-Kdo4*P*	-	-	1.91/2.09	4.44	4.18	3.82	3.68	3.81/3.53
	-	-	*34.4*	*69.7*	*72.2*	*73.2*	*71.4*	*63.2*
	-	-	-	−0.70	-	-	-	-

## Supplementary Materials

The following are available online at www.mdpi.com/1660-3397/15/7/201/s1. Figure S1: Chemical structure of the *H. lacunaris* TB21 R-LPS core oligosaccharide; Figure S2: MALDI MS/MS analysis of the penta-acylated lipid A species at *m/z* 1399.7 from *H. lacunaris* TB21 R-LPS. Fragment assignments are indicated. The double bond position is tentative and remains to be defined; Figure S3: MALDI MS/MS analysis of the tetra-acylated lipid A species at *m/z* 1245.6 from *H. lacunaris* TB21 R-LPS. Fragment assignments are indicated. The double bond position is tentative and remains to be defined; Figure S4: MALDI MS/MS analysis of the tri-acylated lipid A species at *m/z* 1047.5 from *H. lacunaris* TB21 R-LPS. Fragment assignments are indicated. The double bond position is tentative and remains to be defined; Figure S5: Assessment of contaminations in R-LPS. Fold of NF-κB activation and IL-8 release upon stimulation of HEK 293 hTLR2 with 1, 10 and 100 ng/mL of *H. lacunaris* TB21 R-LPS for 6 h and 18 h respectively. Hexa-acylated *E. coli* LPS and Pam3CSK4 were used as controls. * *p* < 0.05, ** *p*<0.01, *** *p* < 0.001 in the Student’s *t*-test (*H. lacunaris* TB21 R-LPS vs. *E. coli* LPS); Figure S6: Cytokine release in BMDMs stimulated with *H. lacunaris* TB21 R-LPS. TNF-α, KC, IL-6 and CCL-5 released by BMDMs after stimulation with 1, 10 and 100 ng/mL of *H. lacunaris* TB21 R-LPS and *E. coli* LPS, measured by ELISA at 18 h. Significant difference between *H. lacunaris* TB21 R-LPS generated values and the corresponding *E. coli* LPS values are indicated (*H. lacunaris* TB21 R-LPS vs. *E. coli* LPS) (* *p* < 0.05; ** *p* <0.01; *** *p* < 0.001).
