# Supplementary figures and images for: The Deep-Sea Polyextremophile Halobacteroides lacunaris TB21 Rough-Type LPS: Structure and Inhibitory Activity towards Toxic LPS
Source: Mar Drugs. 2017 Jun 27;15(7):201. doi: 10.3390/md15070201 (PMC5532643; doi:10.3390/md15070201)

Figure S1

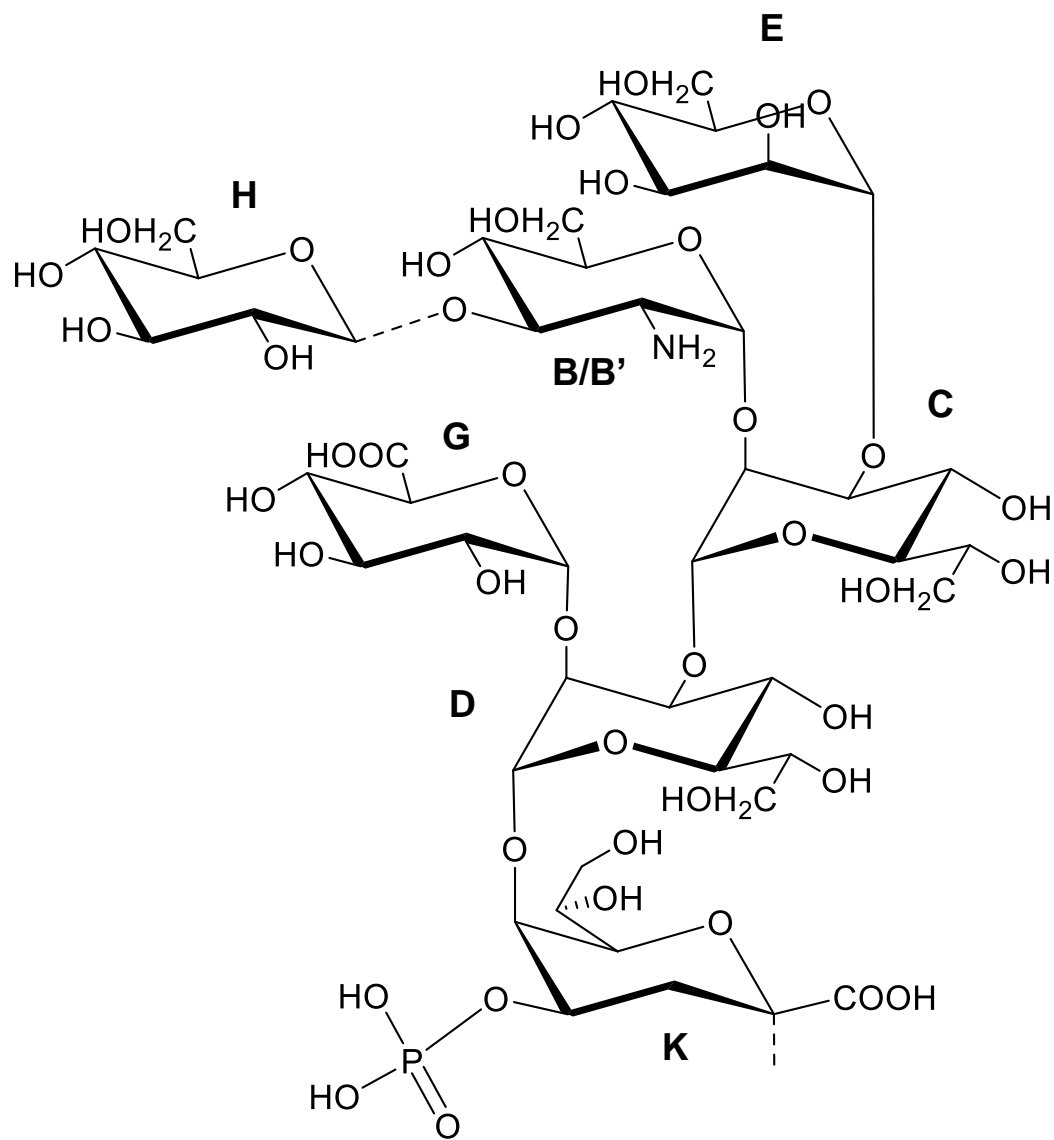

Figure S2

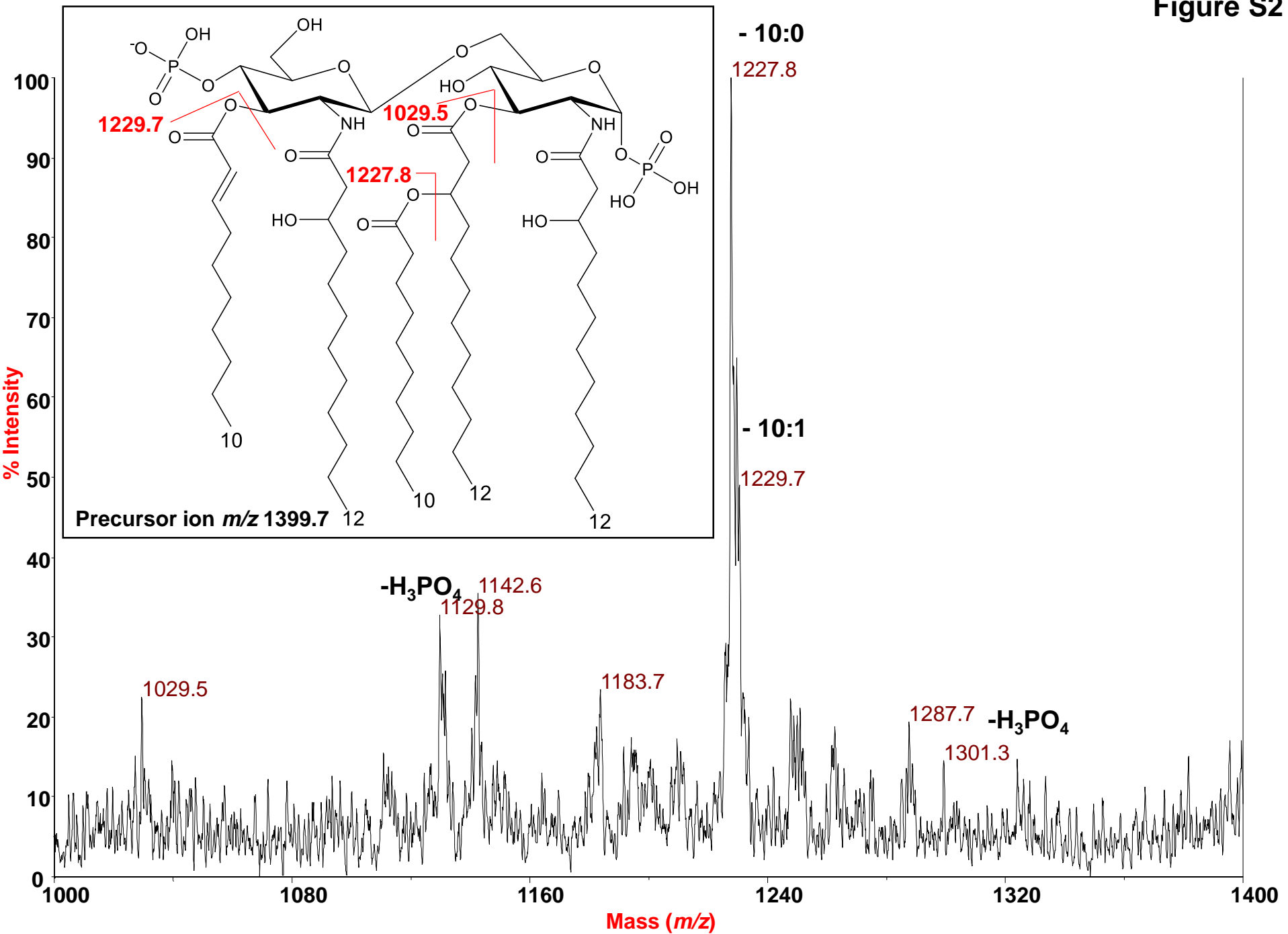

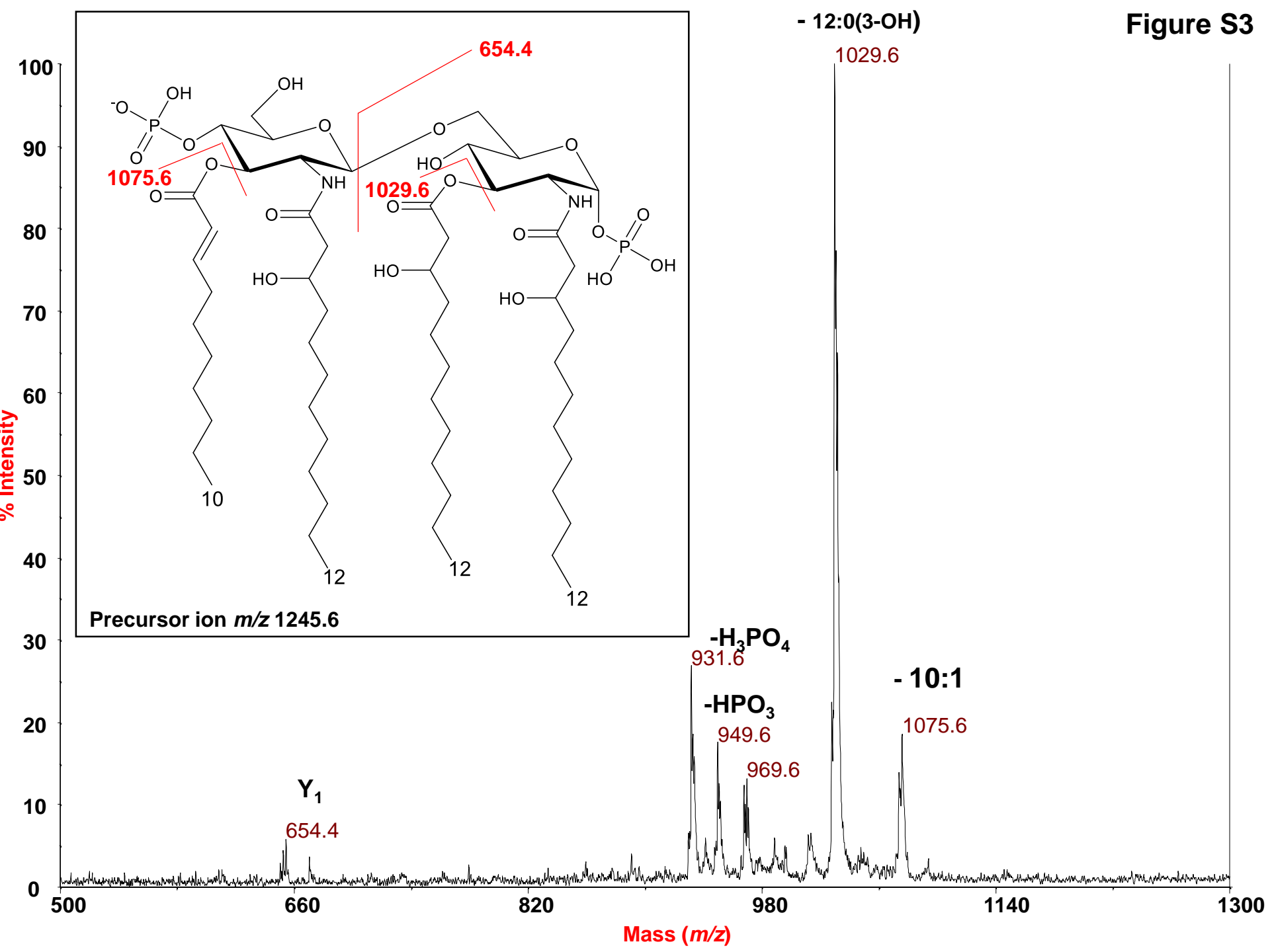

Figure S4

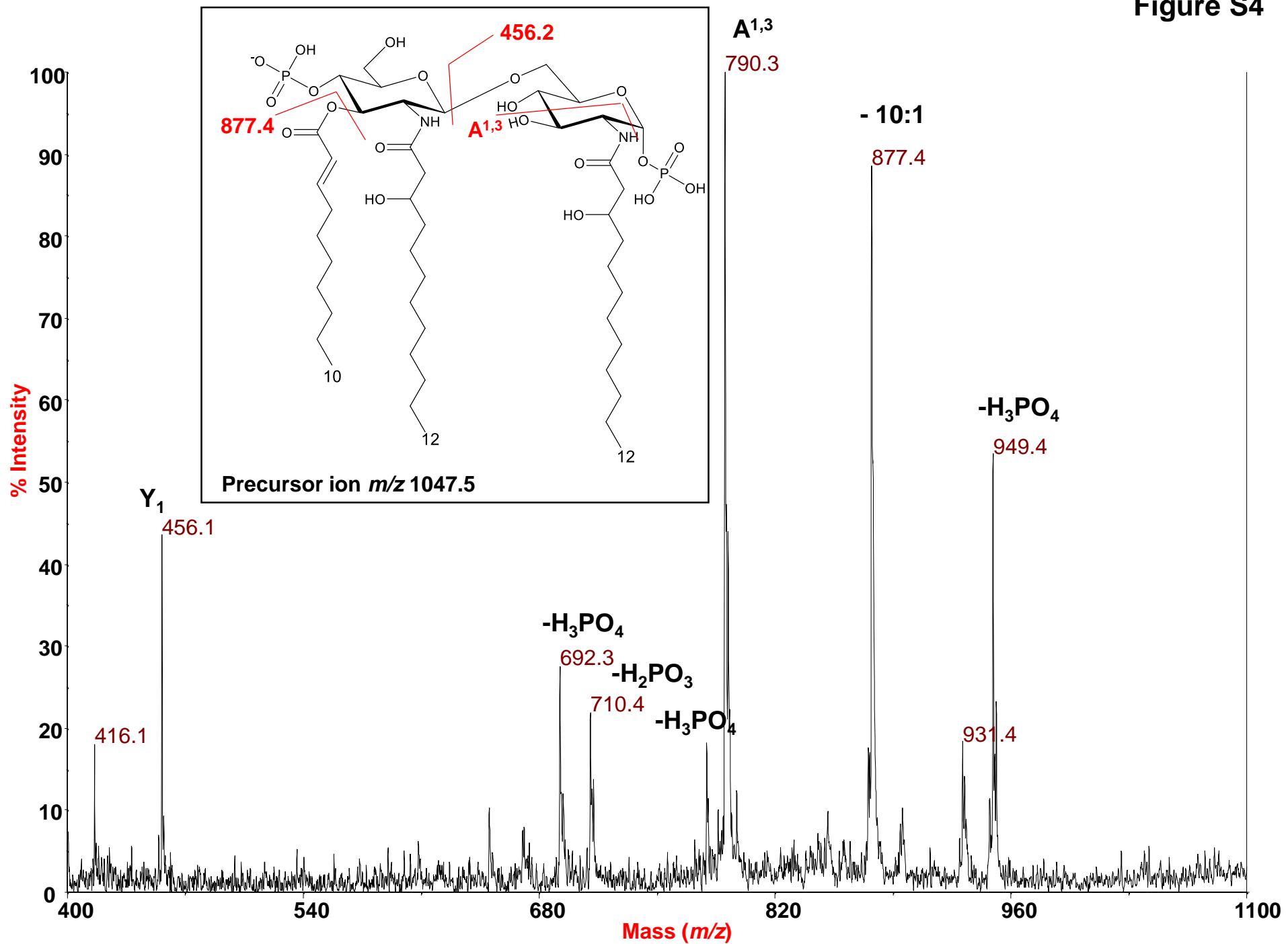

Figure S5

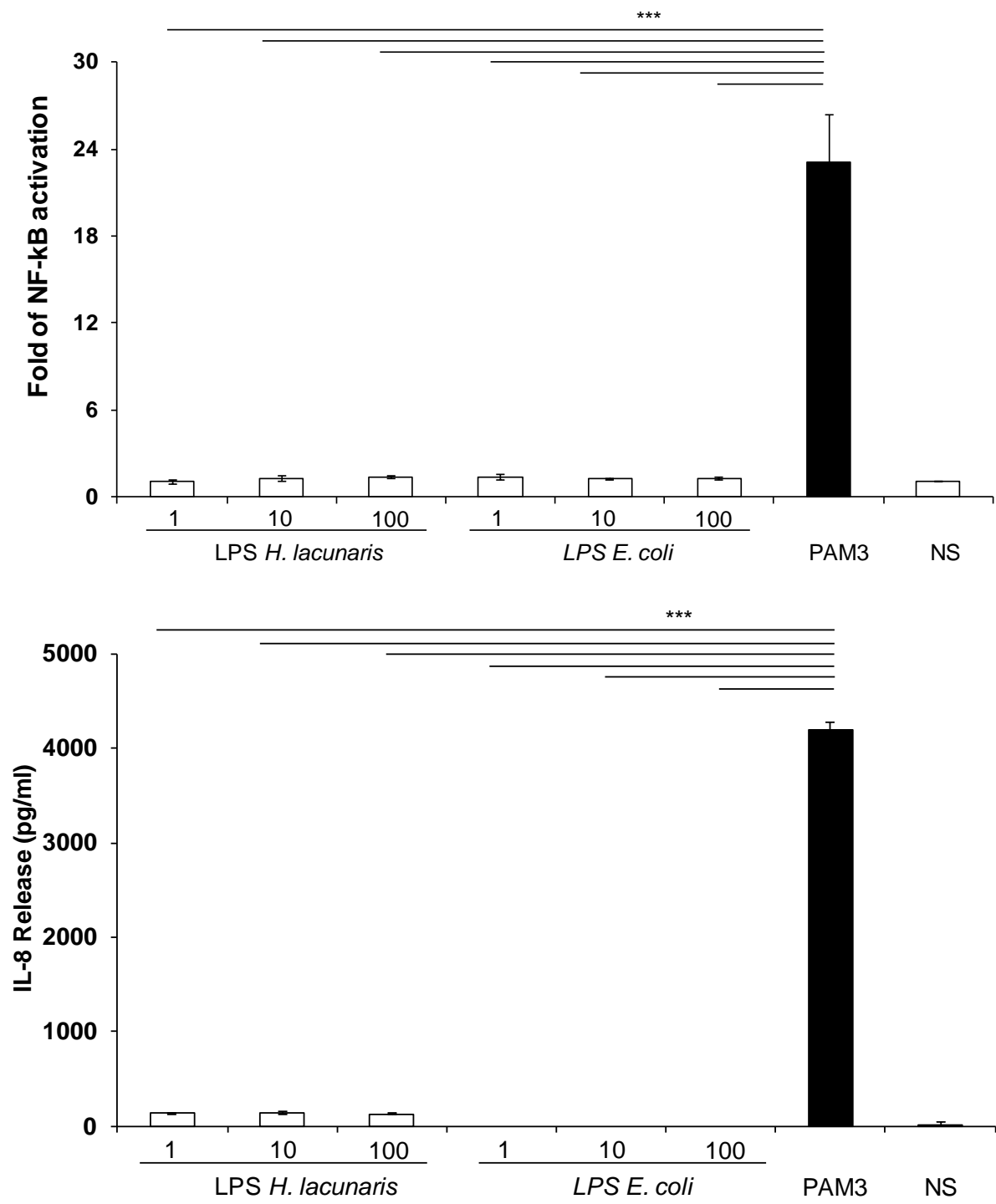

Figure S6

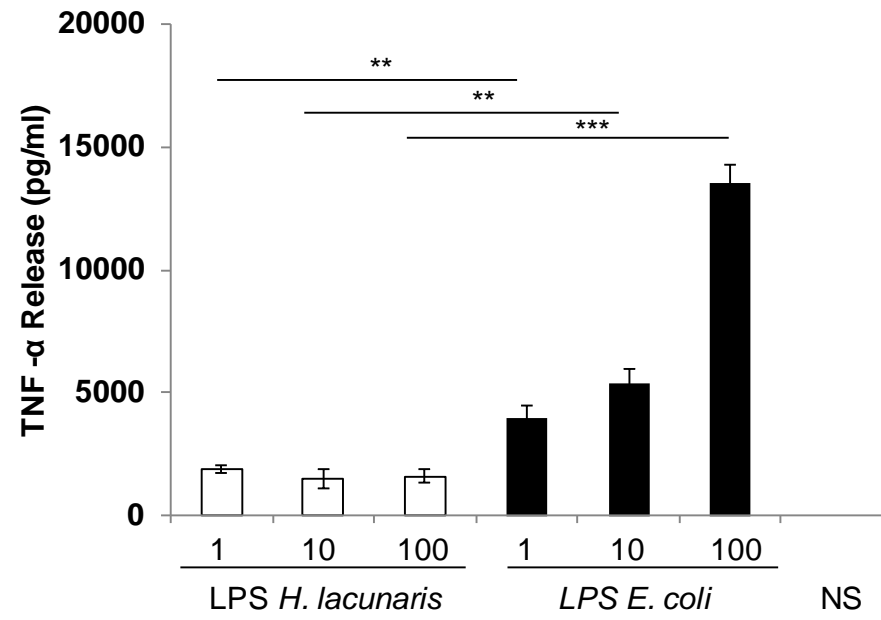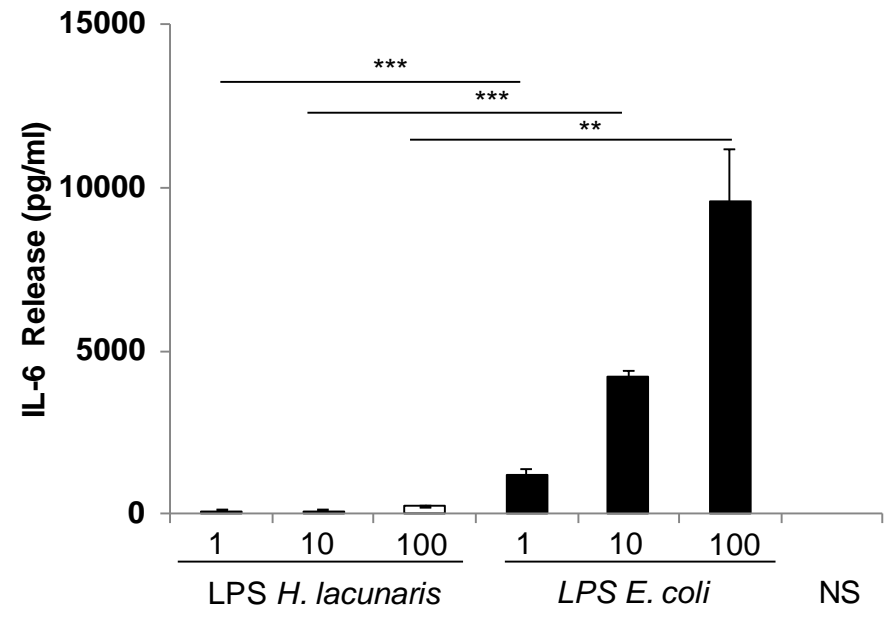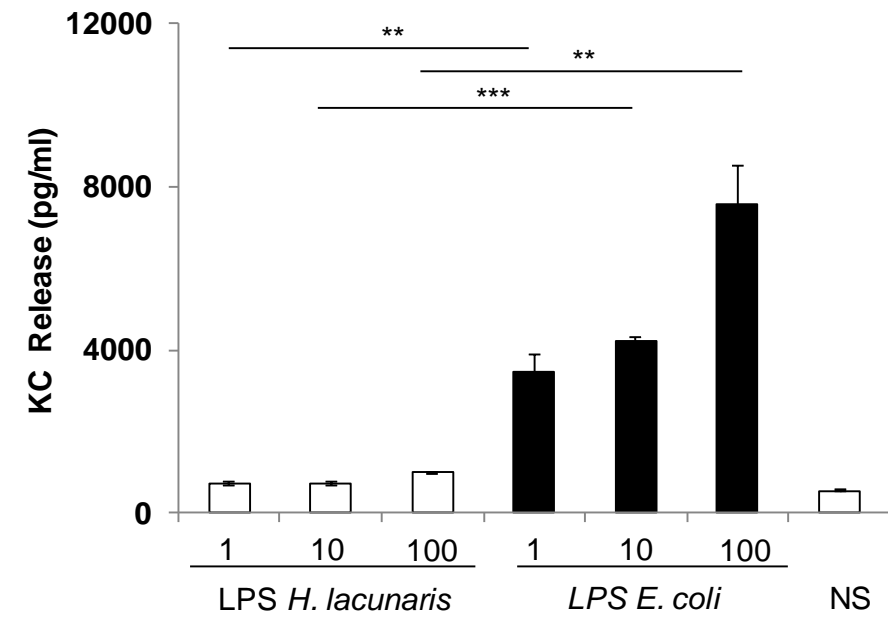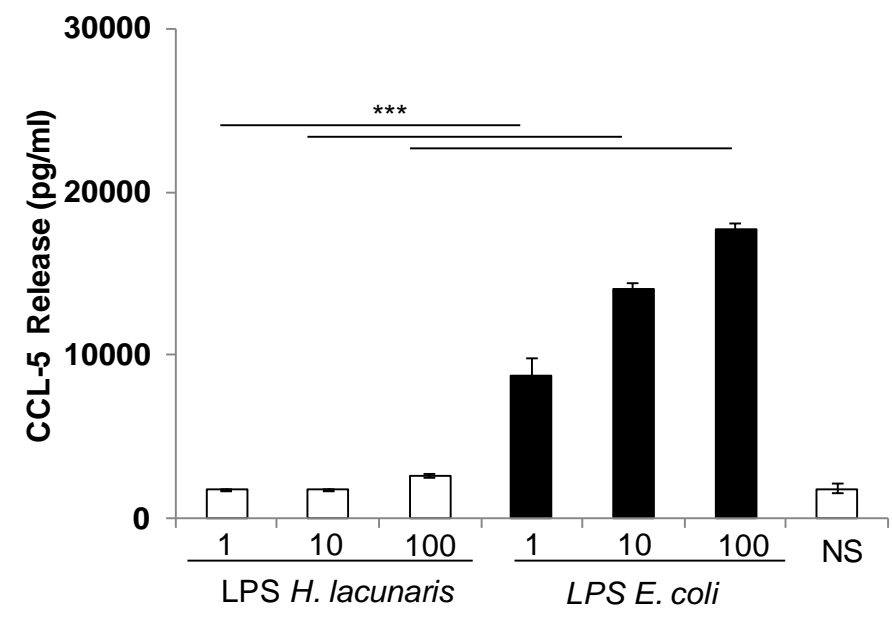

Supplement: Supplementary file 1 [file marinedrugs-15-00201-s001.pdf]
